# A Systematic Review of the Effective Dose of Intrathecal Ropivacaine for Cesarean Section

**DOI:** 10.7759/cureus.91480

**Published:** 2025-09-02

**Authors:** Foteini Vakiarou, Agathi Karakosta, Panagis M Lykoudis, Paraskevi Matsota

**Affiliations:** 1 2nd Department of Anesthesiology, University Hospital Attikon, National and Kapodistrian University of Athens, Athens, GRC; 2 Department of Anesthesiology and Postoperative Intensive Care, University Hospital of Ioannina, University of Ioannina, Ioannina, GRC; 3 4th Department of Surgery, University Hospital Attikon, National and Kapodistrian University of Athens, Athens, GRC

**Keywords:** cesarean section (cs), intrathecal, intrathecal ropivacaine, neuraxial anesthesia, ropivacaine, subarachnoid, subarachnoid space, subdural

## Abstract

Ropivacaine is a frequently used agent in neuraxial anesthesia for cesarean section (C-section). The optimal dose has not been determined yet, and reported doses vary significantly. Higher doses are associated with higher success of anesthesia but also higher rates of adverse effects, such as hypotension. This study aimed to critically appraise current literature in order to determine the optimal dose of ropivacaine, as well as to highlight gaps in current knowledge. Systematic review of comparative studies, without an attempt at meta-analysis due to expected heterogeneity in examined arms. Medical Literature Analysis and Retrieval System Online (MEDLINE), Scopus, and Web of Science were accessed to look for pertinent studies written in English, regardless of time of publication. Included studies compared groups with at least two different doses of ropivacaine for neuraxial anesthesia for C-section in adults. Ten randomized control trials fulfilled eligibility criteria and included a total of 756 patients. Examined doses varied significantly, with 4 mg being the lowest and 25 mg being the highest. The success rate of neuraxial anesthesia ranged from 8.3% to 100%, with this extensive variance being attributed to significant heterogeneity in doses, the definition of success, and methods of measuring it. The effective dose for 50% of patients (ED50) ranged from 6.1 mg to 16.7 mg, while the effective dose for 95% of patients (ED95) ranged from 11.4 mg to 26.8 mg. Quality assessment of studies revealed no significant bias. Current literature on the optimal dose of ropivacaine consists of average and high-quality studies. Those ranked as best describe an ED50 of 8 to 10 mg and an ED95 of 12 to 15 mg. However, the large heterogeneity of tested doses and measures needs to be highlighted, as well as the lack of representation of patients at the upper normal of height.

## Introduction and background

Cesarean section (C-section) is one of the most common obstetric procedures, with an implementation of over 20% worldwide and up to 40% in certain areas of the world [1]. The effectiveness of C-sections is reflected in their ability to reduce perinatal morbidity and mortality in high-risk pregnancies [2, 3]. C-sections are generally considered safe; however, they carry potential risks and complications, including infection, hemorrhage, and complications from anesthesia [4]. A key component in a successful C-section is the administration of anesthesia. Certain parameters are considered for the administration of anesthesia in C-sections, including the route of administration and the agents used. While the first of these parameters has been adequately studied, and the combined spinal-epidural technique has prevailed [5], questions still remain with regard to preferred agents, doses, and combinations. 

Ropivacaine, a long-acting amide local anesthetic, offers adequate sensory and motor block for surgical procedures while minimizing systemic toxicity [6]. Studies have demonstrated that lower doses of intrathecal ropivacaine (10 to 15 mg) can achieve effective anesthesia, significantly reducing the risk of complications associated with higher doses [7]. The safety of intrathecal ropivacaine is well-documented, with a lower incidence of adverse effects such as hypotension and fetal heart rate abnormalities compared to other anesthetics, like bupivacaine [8]. However, potential complications include post-dural puncture headache, transient neurological symptoms, and the rare possibility of neurological injury [9]. Intrathecal ropivacaine is increasingly used as an anesthetic for cesarean sections due to its effectiveness and safety profile. Overall, intrathecal ropivacaine is an effective and safe option for spinal anesthesia in cesarean deliveries, providing reliable analgesia while minimizing risks. Ongoing research is essential to refine dosing guidelines and optimize patient outcomes.

The present study aimed to analyze current literature on the effective dose of intrathecal ropivacaine in C-sections, as well as to highlight clinically relevant topics for further research.

## Review

Materials and methods

Literature Search

The present study was conducted according to the Preferred Reporting Items for Systematic Reviews and Meta-Analyses (PRISMA) guidelines [10]; the corresponding checklist is attached as Appendix 1, and the protocol of the study was registered with the International Prospective Register of Systematic Reviews (PROSPERO) (ID: CRD42025640561). A literature search was conducted on three databases, namely Medical Literature Analysis and Retrieval System Online (MEDLINE), Scopus, and Web of Science. The keywords “ropivacaine,” “intrathecal,” and “cesarean” were looked for in titles and/or abstracts of scientific articles. Inclusion criteria consisted of comparative studies (regardless of prospective or retrospective design) comparing different doses of intrathecal ropivacaine in adult C-sections. Exclusion criteria consisted of articles written in languages other than English and secondary studies (such as systematic reviews, meta-analyses, and case series). Yielded studies were assessed through title and abstract for pertinence, and full texts were sought for those articles deemed relevant. Full texts were then studied to finally decide on the inclusion of articles in the present study. Relevant secondary studies, as well as references of the aforementioned articles, were also screened for identifying pertinent articles that could have been missed in the literature search process. The above procedure was carried out by two researchers independently. In case of disagreement, an attempt was made for discussion and consensus; otherwise, a third researcher provided a final decision.

Data Extraction

Upon determination of the final list of articles for analysis, data extraction was conducted independently by two researchers on a pre-agreed proforma (Microsoft Excel, Microsoft Corporation, Redmond, WA, USA). Extracted parameters consisted of the first author’s name, the year of publication, study design, overall number of patients, compared arms, number of patients per arm, age of patients per arm, American Society of Anesthesiologists (ASA) score per arm, patients’ weight per arm, patients’ height per arm, definition of successful anesthesia, rate of successful anesthesia per arm, effective dose for 50% of patients (ED50), effective dose for 95% of patients (ED95), assessment of sensory block per arm, assessment of motor block per arm, development of hypotension as the absolute number of patients per arm, and mean dose of required vasopressors per arm. As in the previous stage, disagreements between the two researchers were initially addressed through discussion for the possibility of consensus or otherwise resolved by a third researcher. 

Literature Appraisal

Critical appraisal of included studies was conducted using the Methodological Index for Non-Randomized Studies (MINORS) criteria [11] and the Cochrane Risk of Bias 2 (RoB 2) criteria [12]. Similar to previous steps, the assessment was conducted independently by two reviewers, and a third researcher resolved any persisting disagreements. 

Results

**Figure 1:**
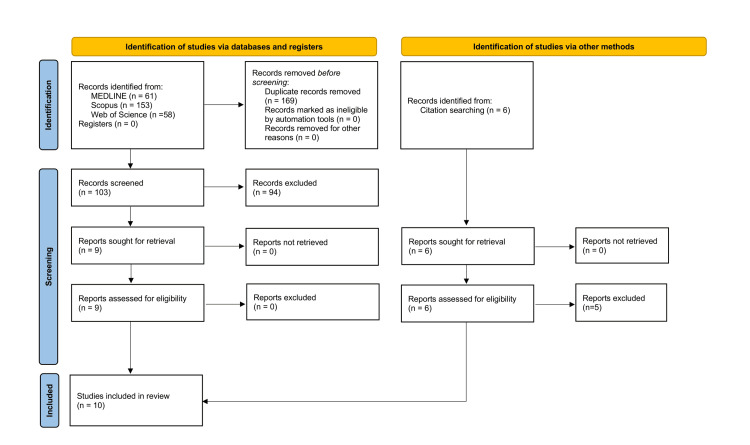
A PRISMA flowchart outlining the methodology steps that were followed to include and exclude the identified studies for the review MEDLINE: Medical Literature Analysis and Retrieval System Online; PRISMA: Preferred Reporting Items for Systematic Reviews and Meta-Analyses

The initial literature search yielded 103 studies. Thirty studies were excluded due to their conduct in other settings, mainly wound infusion-related analgesia. Nineteen studies involved secondary studies and case reports. Seventeen studies compared ropivacaine with bupivacaine, 16 studies examined the effect of a certain dose of ropivacaine within combinations of different agents, and 12 studies only performed a sequential dose analysis, without comparing specific arms. Therefore, nine studies were deemed suitable for analysis [13-21]. One additional study was identified as suitable for analysis through screening references and pertinent secondary studies [22]. Eventually, 10 studies were included in the analysis. The aforementioned process is depicted in Figure 1.

The year of publication ranged from 2001 to 2024, covering thus a span of 23 years [15,21]. All included studies were prospective, randomized trials and included patients with an ASA score of I or II. In two studies, a comparison of different doses of ropivacaine was also conducted between two distinct groups, namely singleton pregnancies and twin pregnancies [16, 21], but for the present study, only data regarding singleton pregnancies were extracted. There was significant variability in terms of the number of arms, with two studies comparing two schemes [14,17], one study comparing three schemes [13], three studies comparing four schemes [15,22], and four studies including four arms [18-21]. The lowest administered dose of ropivacaine was 4 mg of 0.1% [14], and the highest was 25 mg [13, 15]. The two largest studies included an overall number of 100 patients [16, 19], while one study had the largest group, consisting of 40 patients [17]. Age was reported in nine studies, but in two of them, only the overall median age was reported [16, 21]. In the remaining seven studies that reported mean age per group, those ranged from 18.6 [17] to 32.5 [18]. The reporting rate of weight and height for the studied individuals was also similar. Mean weight ranged from 66.3 kg [21] to 79.8 kg [13]. Median height ranged from 159 cm [20] to 166 cm [14]. Demographics of patients in the included studies are presented in Table 1. 

**Table 1:** Demographics of patients in the studies included for analysis ^†^hyperbaric ropivacaine / scarred uterus; •plus phenylephrine IV; ~The study included two groups of patients, singleton and twin pregnancies; this table reports data on singleton pregnancies; RCT: randomized controlled trial; NA: no quantitative report

Author	Year	Design	Arms/Dose of intrathecal ropivacaine (+ adjuncts where reported)	Sample size per arm (number of patients)	Age (median in years)	Weight (median in kg)	Height (median in cm)
Khaw et al. [15]	2001	RCT	10mg vs. 15mg vs. 20mg vs. 25mg	12 vs. 20 vs. 20 vs. 20	NA	NA	NA
Camorcia et al. [14]	2004	RCT	Initial dose of 4mg 1% vs. 0.1%	27 vs. 27	27 vs. 28	73 vs. 71	164 vs. 166
Chen et al. [22]	2006	RCT	10.5mg vs. 12mg vs. 13.5mg vs. 15mg	15 vs. 15 vs. 15 vs. 15	28.79 vs. 29.53 vs. 29.60 vs. 28.47	70.39 vs. 68.57 vs. 68.87 vs. 66.43	160.86 vs. 159.20 vs. 160.00 vs. 161.01
Qian et al. [17]	2008	RCT	10mg (+5μg sufentanil) vs. 15mg	40 vs. 40	18.6 vs. 29.9	70.4 vs. 71.2	160 vs. 161
Xiao et al. [18]	2015	RCT	6mg vs. 8mg vs 10mg vs. 12mg vs. 14mg† / (+5μg sufentanil in all arms)	15 vs. 15 vs. 15 vs. 15 vs. 15	32.3 vs. 29.5 vs. 29.3 vs. 31.1 vs. 32.5	68.6 vs. 67.0 vs. 68.3 vs. 67.5 vs. 68.0	160.7 vs. 161.2 vs. 163.0 vs. 163.1 vs. 160.3
Zheng et al. [20]	2015	RCT	7.5mg vs. 9mg vs. 10.5mg vs. 12mg	20 vs. 20 vs. 20 vs. 20	28 vs. 28 vs. 29 vs. 30	70 vs. 68 vs. 70 vs. 69	162 vs. 160 vs. 161 vs. 159
Ateser and Kayacan [13]	2017	RCT	15mg vs. 20mg vs. 25mg	20 vs. 20 vs. 20	29.05 vs. 30.70 vs. 27.00	79.8 vs. 78.00 vs. 74.75	163.15 vs. 164.65 vs. 163
Xu et al. [19]	2018	RCT	7mg vs. 9mg vs. 11mg vs. 13mg vs. 15mg• / (+5μg sufentanil in all arms)	20 vs. 20 vs. 20 vs. 20 vs. 20	26 vs. 26 vs. 25 vs. 26 vs. 25	73 vs. 70 vs. 71 vs. 70 vs. 72	161 vs. 160 vs. 163 vs. 162 vs. 160
Mei et al. [16]	2020	RCT	9.5mg vs. 11mg vs. 12.5mg vs. 14mg vs. 15.5mg ~	20 vs. 20 vs. 20 vs. 20 vs. 20	31.5	68.2	159.7
Zhu et al. [21]	2024	RCT	10mg vs. 12mg vs. 14mg vs. 16mg vs. 18mg~	15 vs. 15 vs. 15 vs. 15 vs. 15	31.6	66.3	160

There was significant heterogeneity regarding the definition of successful anesthesia. Two studies did not provide a clear definition [13, 17], while one study used a combination of Bromage and Hammersmith Functional Motor Scale (HFMS) scores [14]. The remaining studies utilized a negative pinprick test at dermatomes ranging from T4 to T7 and at a mean time distance from initiation of anesthesia varying from 10 minutes [19-21] to 30 minutes [15]. At these intervals, and given the wide range of doses of ropivacaine, the success rate ranged from 8.3% [15] to 100% [18]. For the aforementioned definitions of successful anesthesia, various mean values of ED50 and ED59 were reported. The former ranged from 6.1 mg [14] to 16.7 mg [15], while the latter ranged from 11.4 mg [20] to 26.8 mg [15]. In both parameters, the aforementioned higher values were derived from the same study that assessed the success of anesthesia at 30 minutes post-initiation [15]. The primary outcomes of included studies are summarized in Table 2.

**Table 2:** Primary outcomes of the included studies ED50: effective dose for 50% of patients; ED95: effective dose for 95% of patients; NA: no quantitative report; *p<0.05; †hyperbaric ropivacaine / scarred uterus; • plus phenylephrine IV; ~The study included two groups of patients, singleton and twin pregnancies; this table reports data on singleton pregnancies; HMFS:  Hammersmith Functional Motor Scale

Author	Year	Arms dose of intrathecal ropivacaine	Definition of success	Success (%)	ED50 (in mg)	ED95 (in mg)
Khaw et al. [15]	2001	10mg vs. 15mg vs. 20mg vs. 25mg	Up to T7 AND no need for supplementation at 30 minutes	8.3 vs. 45 vs. 70 vs. 90	16.7 (14.1-18.8)	26.8 (23.6-34.1)
Camorcia et al. [14]	2004	Initial dose of 4mg 1% vs. 0.1%	Bromage or HMFS >0 at 5 minutes	NA	6.1 (5.1-7.1) vs. 9.1 (7.8-10.3)*	NA
Chen et al. [22]	2006	10.5mg vs. 12mg vs. 13.5mg vs. 15mg	Up to T7 AND no need for supplementation at 20 minutes	40 vs. 66.7 vs. 86.7 vs. 93.3	10.37 (5.23-11.59)	15.39 (13.81-23.59)
Qian et al. [17]	2008	10mg vs. 15mg	NA	NA	NA	NA
Xiao et al. [18]	2015	6mg vs. 8mg vs. 10mg vs. 12mg vs. 14mg†	Up to T4 AND no need for supplementation at 15 minutes	20 vs. 53 vs. 80 vs. 93 vs. 100	8.28 (2.28-9.83)	12.24 (10.53-21.88)
Zheng et al. [20]	2015	7.5mg vs. 9mg vs. 10.5mg vs. 12mg	Up to T7 AND no need for supplementation at 10 minutes	25 vs. 70 vs. 90 vs. 95	8.4 (4.0-9.8)	11.4 (9.7 - 13.9)
Ateser and Kayacan [13]	2017	15mg vs. 20mg vs. 25mg	NA	NA	NA	NA
Xu et al. [19]	2018	7mg vs. 9mg vs.11mg vs. 13mg vs. 15mg•	Up to T5 AND no need for supplementation at 10 minutes	*Higher for groups 4 and 5	9.9 (9.0-10.7)	15.2 (13.5-18.8)
Mei et al. [16]	2020	9.5mg vs. 11mg vs. 12.5mg vs. 14mg vs. 15.5mg ~	Up to T6 AND no need for supplementation at 10 minutes	30 vs. 40 vs. 65 vs. 80 vs. 90	11.2 (10.2-12.0)	15.7 (14.4-18.3)
Zhu et al. [21]	2024	10mg vs. 12mg vs. 14mg vs. 16mg vs. 18mg~	Up to T6 AND no need for supplementation at 10 minutes	NA	9.9 (7.2-11.5)	NA

Two studies did not provide a clear assessment of sensory anesthesia [14, 16], while one study reported this parameter in terms of time taken for anesthesia to retreat by at least two dermatomes, demonstrating a statistically significantly longer time for the group with the higher dose in that study, as well as in the present review [13]. The remaining studies reported this secondary outcome in terms of the median dermatome at which sensory anesthesia was achieved at a certain time point for each study, yet it varied in the group of presently analyzed studies, without any statistically significant differences reported. Respective levels varied from T3 [15] to T7 [19-21]. Heterogeneity was even higher in the reporting of motor anesthesia. Among the six studies that presented relevant data, most reported the time to achieve a satisfactory Bromage score, while one reported the mean duration [18]. Three studies reported statistically significant differences, two of them demonstrating shorter onset for the arm with the higher dose [14, 17], and one reported longer duration for the arms with higher doses [18]. In terms of hypotension, one study did not provide any data [14]; one study did not provide details but mentioned no statistically significant difference [15]; and among the remaining studies that provided more detailed information on this parameter, one reported a statistically significantly higher rate of up to 55% in the group that received a higher dose of ropivacaine [17], while one reported a lower chance of hypertension in the group that received a higher dose of ropivacaine [22]. Five studies did not report on the need for vasopressors, one study did not report quantitative data but mentioned no significant difference [15], and among the four studies that actually presented details on this parameter, three of them demonstrated statistically significantly higher needs in the group of patients that received a higher dose of ropivacaine [17, 18]. The data on secondary outcomes are summarized in Table 3.

**Table 3:** Secondary outcomes of analyzed studies NA: no quantitative report; NS: reported as non-statistically significant; *p<0.05; †hyperbaric ropivacaine/scarred uterus; ‡Onset of motor anesthesia; §Duration of motor anesthesia; °Time till regression by two dermotomes; •plus phenylephrine IV; ~The study included two groups of patients, singleton and twin pregnancies; this table reports data on singleton pregnancies

Author	Year	Arms dose of intrathecal ropivacaine	Sample size per arm	Sensory	Motor	Hypotension (number of patients)	Administration of vasopressors
Khaw et al. [15]	2001	10mg vs. 15mg vs. 20mg vs. 25mg	12 vs. 20 vs. 20 vs. 20	T5 vs. T3 vs. T3 vs. T3	NA	NS	NS
Camorcia et al. [14]	2004	Initial dose of 4mg 1% vs. 0.1%	27 vs. 27	NA	6.1mg vs. 9.1mg‡*	NA	NA
Chen et al. [22]	2006	10.5mg vs. 12mg vs. 13.5mg vs. 15mg	15 vs. 15 vs. 15 vs. 15	T6 vs. T6 vs. T5 vs. T5	2.87 vs. 2.8 vs. 2.87 vs. 2.93‡	6 vs. 3 vs. 2 vs. 0*	NA
Qian et al. [17]	2008	10mg vs. 15mg	40 vs. 40	T4 vs. T4	4.6min vs. 2.9min‡*	20% vs. 55%*	6mg vs. 9 mg of ephedrine*
Xiao et al. [18]	2015	6mg vs. 8mg vs. 10mg vs. 12mg vs. 14mg†	15 vs. 15 vs. 15 vs. 15 vs. 15	T4 vs. T4 vs. T4 vs. T4 vs. T4	38min vs. 55min vs. 68min vs. 116min vs. 125min§*	3 vs. 5 vs. 4 vs. 6 vs 8	0 vs. 0 vs. 0 vs. 0 vs. 40μg of phenylephrine*
Zheng et al. [20]	2015	7.5mg vs. 9mg vs. 10.5mg vs. 12mg	20 vs. 20 vs. 20 vs. 20	T7 vs. T5 vs. T5 vs. T5	7.5min vs. 5min vs. 5min vs. 5min‡	3 vs. 4 vs. 5 vs. 7	5mg vs. 5mg vs. 5mg vs. 5mg of ephedrine
Ateser and Kayacan [13]	2017	15mg vs. 20mg vs. 25mg	20 vs. 20 vs. 20	127.8min vs. 141.40 vs. 163.10°*	1.4 vs. 1.6 vs. 1.45‡	8 vs. 8 vs. 10	Higher need for ephedrine in group 3*
Xu et al. [19]	2018	7mg vs. 9mg vs. 11mg vs. 13mg vs. 15mg•	20 vs. 20 vs. 20 vs. 20 vs. 20	T7 vs. T6 vs. T5 vs. T5 vs T4	NA	3 vs. 2 vs. 2 vs. 4 vs 3	NA
Mei et al. [16]	2020	9.5mg vs. 11mg vs. 12.5mg vs. 14mg vs. 15.5mg ~	20 vs. 20 vs. 20 vs. 20 vs. 20	NA	NA	37	NA
Zhu et al. [21]	2024	10mg vs. 12mg vs. 14mg vs. 16mg vs. 18mg~	15 vs. 15 vs. 15 vs. 15 vs. 15	T7	NA	46	NA

As far as quality assessment is concerned, Table 4 presents the MINORS scoring of included studies.

**Table 4:** Assessment of included studies according to the MINORS criteria MINORS: Methodological Index for Non-Randomized Studies

Reference	Study aim	Consecutive patients	Data collection Methodology	Reported Endpoints	Outcome evaluation bias	Equivalent groups	Statistical methods	Follow-up period	Follow-up loss	Overall score
Khaw et al. [15]	2	1	2	2	2	2	2	2	2	17
Camorcia et al. [14]	2	1	2	2	2	2	1	2	2	16
Chen et al. [22]	2	1	2	2	2	2	1	2	2	16
Qian et al. [17]	2	1	2	2	2	1	2	2	2	16
Xiao et al. [18]	2	1	2	2	2	2	2	2	2	17
Zheng et al. [20]	2	1	2	2	2	2	1	2	2	16
Ateser and Kayacan [13]	2	1	2	2	2	2	1	2	2	16
Xu et al. [19]	2	1	2	2	2	2	2	2	2	17
Mei et al. [16]	2	1	2	2	2	2	2	2	2	17
Zhu et al. [21]	2	1	1	2	2	2	2	2	2	17

**Figure 2:**
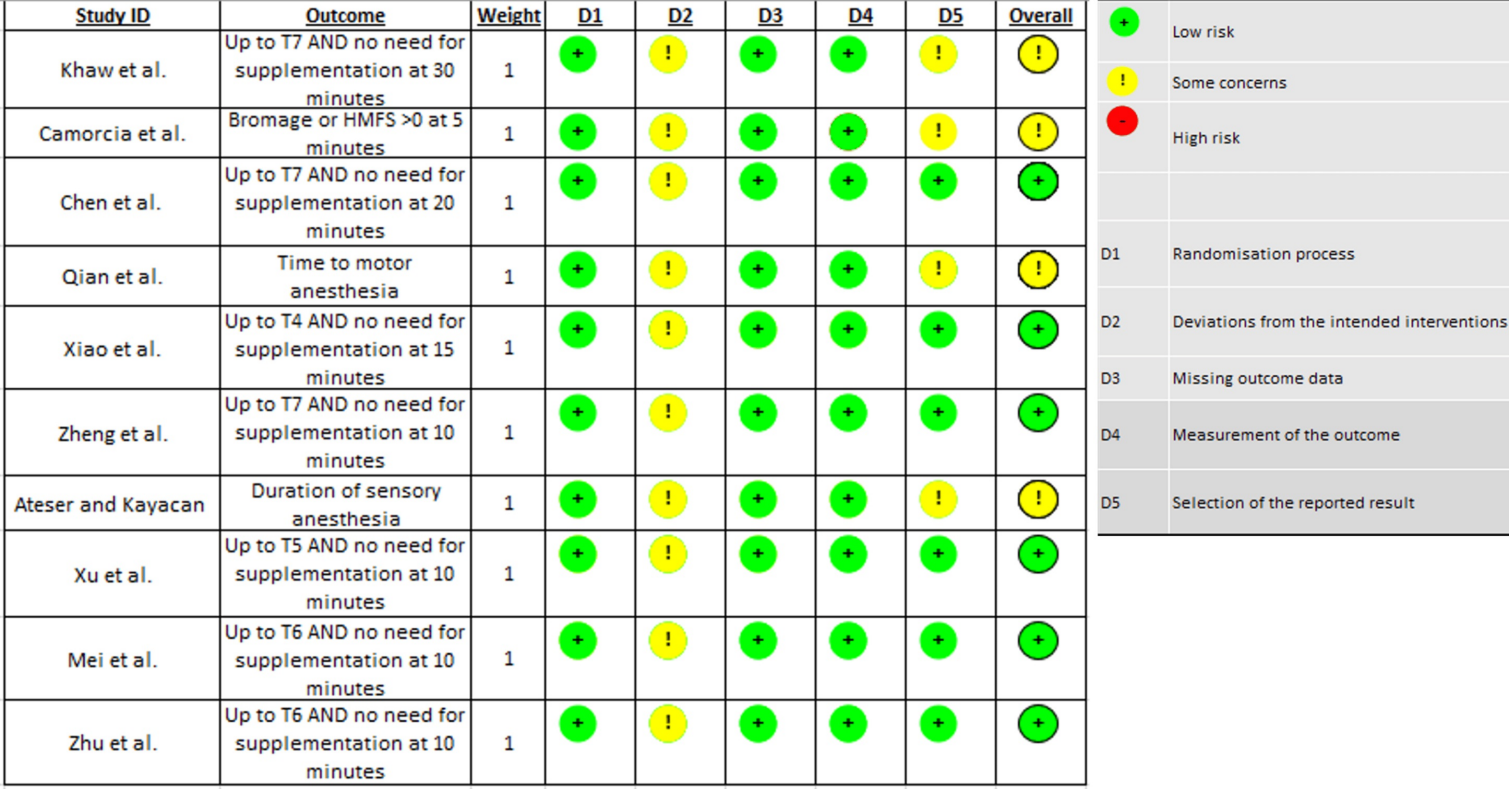
Assessment of included studies according to RoB 2 criteria RoB 2: Risk of Bias 2; HMFS: Hammersmith Functional Motor Scale

All studies missed one point due to not clearly mentioning that all consecutive patients were considered for eligibility, and all eligible patients were included in the respective study. One study missed one point, as it was not clear if treatment was equivalently delivered between the two groups and if the groups were actually similar in terms of baseline characteristics [17]. Four studies missed one point because they reported using parametric tests only, without providing evidence of normal distribution of their data, especially given the relatively small number of patients [20,22]. However, the overall scores were quite high, ranging from 16 to 17. In terms of the RoB 2 criteria, no study was identified as high risk for bias (Figure 2).

Dimension 2, deviation from the intended interventions, was the reason why some concerns for bias were raised for all included studies. The protocol of response to inadequate sensory or motor anesthesia was not explicit in any study, and therefore, interventions cannot be considered entirely reproducible and unbiased. For four studies, some concerns were also raised with regard to Dimension 5, i.e., selection of reported outcomes. For one of the studies, the interval of 30 minutes for the assessment of sensory anesthesia level is considered too long [15]. For the remaining three, the definition of the outcome was not entirely clear; motor anesthesia assessment can be subjective, and the duration of anesthesia can be affected by many unreported parameters. Therefore, these four studies received an overall assessment of raising some concerns due to concerns in two different dimensions [13-17].

Discussion

Spinal anesthesia induction with ropivacaine is both effective and safe in C-section procedures. To date, only a single meta-analysis including eight randomized trials has been published, comparing ropivacaine against bupivacaine in C-sections [23]. The present systematic review included studies examining different doses of ropivacaine prior to C-section. The literature search yielded 10 randomized controlled trials deemed suitable for inclusion. With the already described dosage discrepancy, ranging from 4 mg to 25 mg, the most common dose administered was 15 mg. Regarding ED50 and ED95, the most commonly reported values were 9.9 mg and 15 mg, respectively. The definition of success was not at all given in two studies, while six of the rest defined it variably, and only Mei et al. [16] and Zhu et al. [21] had a similar definition of successful sensory anesthesia, up to T6 without supplementation at 10 minutes. Progressive dose-dependent escalation of time-to-successful anesthesia was recorded only by Mei et al. [16]. Despite the lack of a uniform definition, T7 was the most commonly used spinal level. The prevalent success rate approached 95% and was achieved with a dosage of 12.5 mg, with a corresponding increase in success rate in cases of increasing dosage. As far as secondary outcomes were concerned, motor anesthesia was not properly reported in half of the studies, as emphasis was given on sensory anesthesia in eight studies. Hypotension was observed in most studies to some extent, significantly by Ateser and Kayacan [13], where the highest dose of ropivacaine was administered amongst all analyzed studies. This is supported by Hong et al., in whose study it has been delineated that high concentrations of ropivacaine through epidural anesthesia cause a significant decrease in the systemic vascular resistance and blood pressure [24].

In a prospective, randomized, double-blind investigation using a dose escalation protocol, the ED50 and estimated ED95 for spinal ropivacaine were 16.7 mg and 26.8 mg, respectively [15]. The importance of assessing the motor block level has also been highlighted, as it has an important role in determining adequacy of spinal anesthesia, given that an incomplete motor block is associated with failed anesthesia [25]. In a meta-analysis by Cossu et al. [26], it was shown that the neuraxial administration of neostigmine significantly reduced local anesthetic consumption, without serious adverse side effects to the mother or the fetus. However, neostigmine may only be recommended for epidural administration since intrathecal administration significantly increases the incidence of maternal nausea and vomiting.

With regard to comparison with the other widely used potent anesthetic agent, bupivacaine, ropivacaine demonstrates advantages, as it is associated with weaker hemodynamic changes, lower duration of sensory and motor block, and fewer side effects, which are important for patient recovery [9]. In a meta-analysis by Malhotra et al., when ropivacaine was compared with bupivacaine, intrathecal ropivacaine was associated with more rapid recovery of motor block despite similar sensory properties and no increase in the rate of conversion to general anesthesia [27]. The authors of this study also suggested this finding as an argument in favor of ropivacaine use in centers where recovery of motor block is a criterion for discharge from the post-anesthesia care unit.

The quality of the included studies was evaluated using both MINORS and RoB 2 criteria. With the MINORS criteria, overall scores were high among all studies, ranging from 16 to 17. According to RoB 2 criteria, six studies were defined as low risk for bias, and four raised some concerns. The most important key points that deducted points were the lack of a detailed protocol in case of inadequate anesthesia, as well as not clearly confirming whether patients were consecutive. Recruiting non-consecutive patients in a study can introduce a significant level of bias. In addition, optimizing the selection of benchmarks and time points of measurements is crucial for the overall implementability of a randomized trial.

In terms of clinical practice, it is safe to conclude that ropivacaine is a well-tolerated agent, with rare adverse events, despite the wide range of tested dosages. Guidelines lack a consensus on a proposed calculation for the required dose, with a view to achieving the most favorable pharmacological effect. Therefore, decision-making is currently based on individual preference. Out of the 10 included studies, Xiao et al. [18] and Xu et al. [19] achieved the highest scores if both systems are considered. In the study of Xiao et al. [18] that was performed in a specific subpopulation with a scarred uterus, 6, 8, 10, 12, or 14 mg of intrathecal hyperbaric ropivacaine was administered, supplemented with 5 μg of sufentanil. Successful spinal anesthesia was defined as a T4 sensory block achieved without the need for epidural supplementation. ED50 and ED95 were 8.2 mg (95% CI: 2.28-9.83 mg) and 12.24 mg (95% CI: 10.53-21.88 mg), respectively [18]. Similarly, in the study by Xu et al., patients were randomly assigned to receive 7, 9, 11, 13, or 15 mg intrathecal hyperbaric ropivacaine, respectively [19]. Prophylactic phenylephrine infusion (50 μg/min) was initiated immediately at the same time as the spinal injection. The ED50 and ED95 for successful anesthesia were 9.9 mg (95% CI, 9.0-10.7 mg) and 15.2 mg (95% CI, 13.5-18.8 mg), respectively, slightly higher than those reported by Xiao et al. Therefore, it can be argued that a dose of 12 to 15 mg could be considered as optimal, with patients at the higher end of the aforementioned range possibly benefiting from the prophylactic administration of phenylephrine. In the context of lower limb surgery, spinal ropivacaine’s ED50 and ED95 for a duration of anesthesia of 50 minutes or less were estimated at 7.6 mg and 11.4 mg, respectively. This demonstrates rough accordance with the findings of the present study and can further support decision-making on the dose of spinal ropivacaine for C-sections [28]. 

In terms of guidance for further research, two main points should be highlighted. The first point would be the need to address several elements of heterogeneity in existing literature, starting from the determination of optimal benchmarks for monitoring successful anesthesia and timepoints at which these should be checked. Ideally, both sensory and motor block quality should be addressed, and therefore, a specific level for the former and a specific assessment tool for the latter should be selected, ideally through a Delphi consensus approach. Further heterogeneity could also be avoided by encouraging studies with fewer arms, more distinct dosage options, and standardized responses to intraoperative anesthesia fluctuation. The second point that should be highlighted through the present study is that most of the included trials were conducted on Asian populations, and this might be the reason why the highest mean height of examined women was 166 cm. Given that height is directly associated with the length of the spinal cord, which in turn is associated with the space and capacity of epidural and subarachnoid spaces, it can be argued that height can be associated with required doses of neuraxial ropivacaine. In this case, findings from studies conducted on populations with a lower mean height might not necessarily be implementable on populations with taller women. In the western world and particularly in northern Europe, this might be the case, and therefore dose assessment should ideally be conducted on these populations too. 

Although all the papers included were randomized controlled trials, the authors of the present review decided against performing a quantitative aggregation of the data in the form of a meta-analysis. The main reason for this decision was the inconsistent definition of the success parameter. In addition, the large date span, the discrepancies between dosage administered, the diverse study arms, and the heterogeneity of primary outcomes would introduce important limitations in such an effort. This heterogeneity could be considered the major shortcoming of the present study; however, despite it, the overall quality of the analyzed studies was good, and findings can be considered aligned.

## Conclusions

In conclusion, current literature consists of good-quality randomized control trials, and the best of those seem to agree that the ED50 and ED95 of neuraxial ropivacaine in C-sections are 8-10 mg and 12-15 mg, respectively, without significant adverse events. However, certain areas for improvement of future research have been highlighted, mainly on determining and aligning optimal measures of successful anesthesia and evaluating the dose of the agent in populations with different somatometric characteristics.

## Figures and Tables

**Figure 3:**
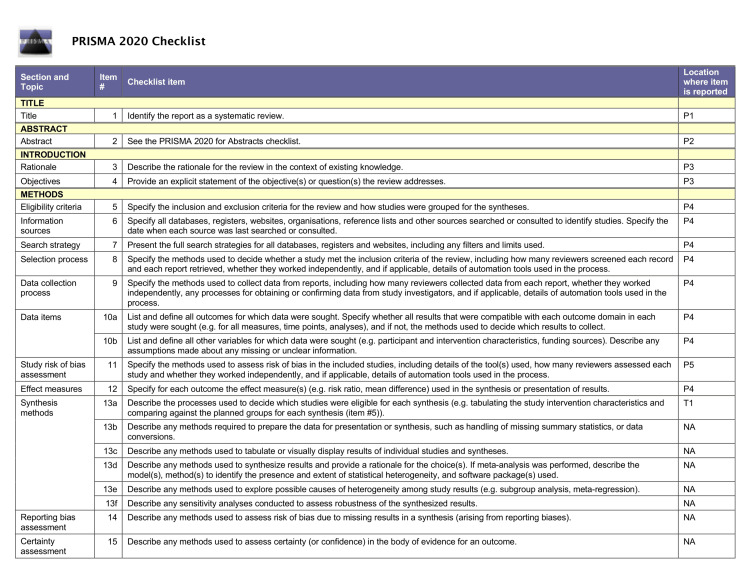
PRISMA 2020 Checklist PRISMA: Preferred Reporting Items for Systematic reviews and Meta-Analyses Reproduced from [10]. This work is licensed under CC BY 4.0.

## Appendices

Appendix A 
